# The Effect of Resistance Training Interventions on ‘The Self’ in Youth: a Systematic Review and Meta-analysis

**DOI:** 10.1186/s40798-019-0205-0

**Published:** 2019-07-03

**Authors:** Helen Collins, Josephine N. Booth, Audrey Duncan, Samantha Fawkner, Ailsa Niven

**Affiliations:** 1 Physical Activity and Health Research Centre, Institute of Sport, Physical Education and Health Sciences, Moray House School of Education and Sport, University of Edinburgh, Edinburgh, UK; 2 Institute of Sport and Exercise, University of Dundee, Old Hawkhill, Dundee, UK; 3 Institute of Education, Community and Society, Moray House School of Education and Sport, University of Edinburgh, Edinburgh, UK

**Keywords:** Resistance training, Children, Adolescents, Self, Strength

## Abstract

**Background:**

There is growing evidence that physical activity (PA) is beneficial for the mental health of young people. One area that has been widely examined is the impact of PA on ‘the self’, which is a term that encompasses a range of specific and related terms (e.g. self-esteem, self-efficacy, self-perceptions). There is evidence that PA is strongly associated with ‘the self’ in childhood and beyond. However, the impact of the specific PA of resistance training (RT) is not yet clear. The purpose of this review was to advance knowledge on the potential of RT for enhancing mental health by examining the effect of RT interventions on ‘the self’ in youth.

**Methods:**

This systematic review followed the PRISMA guidelines (PROSPERO registration number CRD42016038365). Electronic literature databases were searched from the year of their inception to October 2018. The search included English language articles that examined the effect of isolated RT on the broad term of ‘the self’ in youth, with participants of school age (5–18 years). Data were extracted using an electronic form by one reviewer with 10% conducted by a second reviewer. The ‘Quality Assessment Tool for Quantitative Studies’ was used to assess the quality and risk of bias and was conducted by two reviewers.

**Results:**

From seven peer-reviewed studies, ten data sets were included exploring seven outcomes related to ‘the self’ in participants aged between 10 and 16 years. Four of these studies (including seven data sets) were combined in a meta-analysis, with results from the remaining three studies reported separately. Significant intervention effects were identified for resistance training self-efficacy (Hedges’ *g* = 0.538, 95% CI 0.254 to 0.822, *P* < 0.001), physical strength (Hedges’ *g* = 0.289, 95% CI 0.067 to 0.511, *P* = 0.011), physical self-worth (Hedges’ *g* = 0.319, 95% CI 0.114 to 0.523, *P* = 0.002) and global self-worth (Hedges’ *g* = 0.409, 95% 0.149 to 0.669, *P* = 0.002). Although not statistically significant, the effect sizes for the remaining three outcomes were body attractiveness (Hedges’ *g* = 0.211, 95% CI − 0.031 to 0.454, *P* = 0.087), physical condition (Hedges’ *g* = 0.089, 95% CI − 0.238 to 0.417, *P* = 0.593) and sport competence (Hedges’ *g* = 0.004, 95% CI − 0.218 to 0.225, *P* = 0.974). There was variable quality of studies, with just two studies being classified as ‘strong’.

**Conclusion:**

This is the first review to synthesise research on the effects of isolated RT interventions on ‘the self’. The findings indicate that RT has a positive impact on some aspects of ‘the self’ in youth. More high-quality studies should be conducted to further investigate this topic. If validated, this type of intervention could have a positive impact on ‘the self’ and ultimately improve the health of individuals not only during childhood but as they progress through life.

## Key Points

- Physical activity guidelines and position statements emphasise the importance of ‘activity to strengthen muscle and bone’. Furthermore, research suggests that resistance training might have an impact on ‘the self’ in youth.
- Resistance training was found to have a positive effect on resistance training self-efficacy, perceived physical strength, physical self-worth, and global self-worth.
- If validated, this type of intervention, as recommended by the UK and WHO physical activity guidelines, could ultimately have a positive impact on ‘the self’ and improve the health of individuals not only during childhood but as they progress through life.

## Background

The positive effects of physical activity (PA) on the health and well-being of youth are well established. The most up to date review of evidence states that appropriate levels of PA contribute to the development of healthy musculoskeletal tissues, a healthy cardiovascular system, and neuromuscular awareness [1]. In addition to the physical benefits, there is growing evidence that PA is beneficial for the mental health of young people, including depression, anxiety and self-esteem [2], and in this respect, one area that has been widely examined is the impact of PA on ‘the self’ [3].

The term ‘the self’ is used in this paper to capture a range of specific terms that, while separate, are related (e.g. self-esteem, self-efficacy, self-perceptions). In this respect, PA researchers have typically defined ‘the self’ as having a multi-dimensional and hierarchical structure. This involves more stable constructs (e.g. self-concept ‘a person’s perception of him or herself’ [4] and self-esteem ‘how an individual feels about their sense of self’ [5]) at the apex of the structure which are influenced by less stable sub-domains (e.g. physical self-perceptions ‘a person’s perceptions of their physical self’ [6]) and further sub-areas (e.g. perceived strength).

There is review-level evidence that provides support for a positive relationship between PA and constructs relating to ‘the self’ in youth [7–10]. Babic et al. [10] included 64 studies in a meta-analysis and found a significant association between physical activity and physical self-concept and its various sub-domains in children and adolescents. Despite some limitations regarding methodological design, collectively, these reviews provide convincing evidence of the beneficial effects of PA on the ‘the self’.

**Fig. 1:**
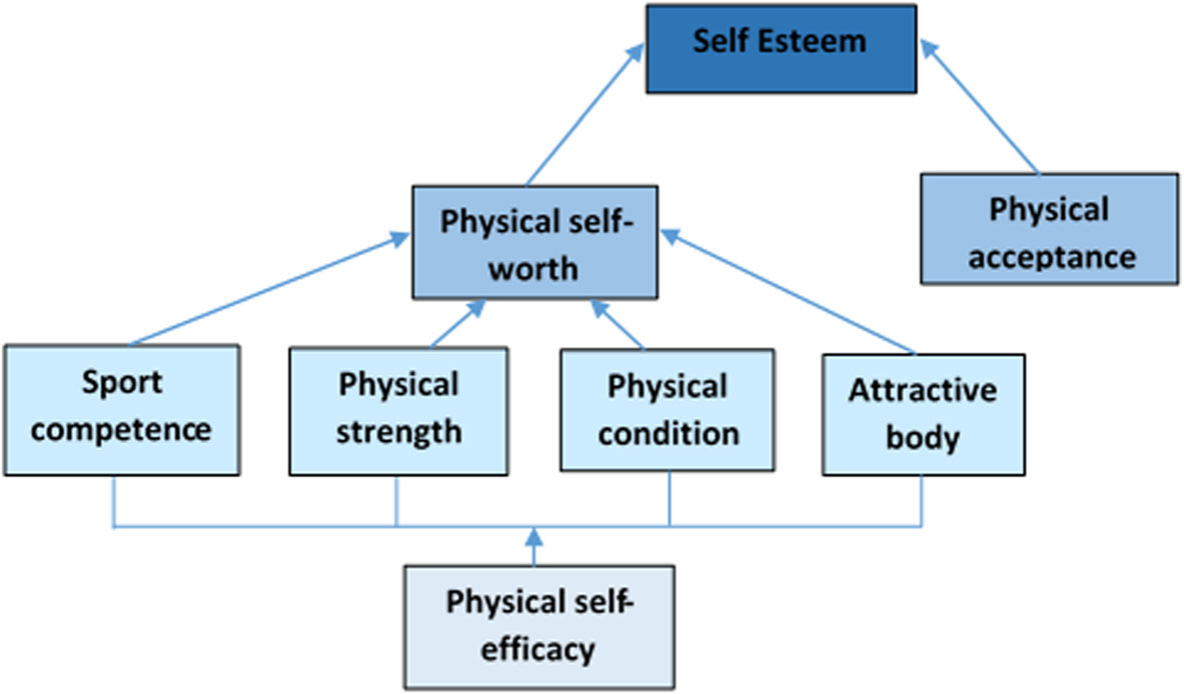
Adaptation of the Exercise and Self-esteem (EXSEM) model [11] (reprinted, by permission from Fox [6])

To explain the mechanism behind the association between PA and ‘the self’, Sonstroem and Morgan [11] proposed the Exercise and Self-esteem Model (EXSEM). Fox [6] updated the EXSEM model by integrating the Physical Self-perception Profile (PSPP) [12] hierarchical structure (Fig. 1). Within this model, it was proposed that physical responses to exercise (as assessed by physical measures) can influence physical self-efficacy via perceptions of what the body can do (e.g. sport competence, physical strength) and how the body looks (e.g. body attractiveness) [5]. Physical self-efficacy is not a reflective description or evaluation of the self but rather a more situation-specific self-assessment of perceived ability [5]. Within this EXSEM model, Sonstroem and Morgan [11] suggest that self-efficacy expectations regarding particular exercise activities will constitute the most immediate and specific self-perception (i.e. at the bottom of the hierarchy). Self-efficacy is viewed to be the most accurate and most influenced by the environment, and over time, these perceptions can feed forward to influence broader perceptions of physical competence and, ultimately, self-esteem [13].

Supporting the EXSEM model, Lubans et al. [14] presented a recent conceptual model through the process of a systematic review that explored the mechanisms underlying the effect of physical activity on mental health outcomes, including ‘the self’. This model proposes that physical activity has an impact on psychosocial factors (i.e. social connectedness, mood and emotions and physical self-perceptions) which ultimately has an effect on global self-esteem.

In general, the youth PA interventions described in research predominantly focus on the aerobic component of the youth PA guidelines. The PA guidelines however also recommend including activities that strengthen the muscle and bone, on at least 3 days a week [15, 16], and key organisations (National Strength and Conditioning Association (NSCA) [17], United Kingdom Strength and Conditioning Association (UKSCA) [18] and The British Association of Sport and Exercise Sciences (BASES) [19]) have developed position statements emphasising why youth should be engaged in resistance training (RT). RT refers to a specialised method of conditioning whereby an individual is working against a wide range of resistive loads in order to enhance health, fitness and performance [18]. A benefit identified in the position statements is the positive effect of RT on ‘the self’ [17–19]. For example, Lubans et al. [20] investigated whether RT might impact on ‘resistance training (RT) self-efficacy’. This is defined as participants’ confidence and beliefs about RT [20]. In the same way that physical self-efficacy represents an immediate and specific measure of self-efficacy related to physical activities, RT self-efficacy represents an immediate and specific measure of self-efficacy related to RT. RT self-efficacy could be an important factor when considering both the benefits of an RT intervention and adherence to such an intervention.

Despite this positive endorsement of RT in both the PA guidelines and respective position statements, the evidence to support a positive effect of RT on ‘the self’ is inconclusive. Although reviews by Ahn and Fedewa [8] and Liu et al. [9] examining the effect of PA on ‘the self’ in youth included RT interventions, the independent role of RT was not considered. Indirect support for the independent positive effect of RT interventions on the self therefore currently comes from studies that demonstrate a positive association between muscular fitness and physical self-perceptions [21]. Specifically, Lubans and Cliff [22] reported evidence of an association between muscular fitness and physical self-perceptions (perceived physical performance and perceived sports competence), overall physical self-worth and global self-esteem in youth. However, due to the cross-sectional nature of these data, it remains difficult to untangle the direction of the effect.

Thus, although there is evidence of an association between muscular fitness and ‘the self’, it remains unclear whether there is a direct effect of RT on ‘the self’. Indeed, in the BASES position statement, it was argued that there has been limited research on the effects of RT on the psychological well-being of children and adolescents, and 14 years later, the position appears to have changed little [19]. The current review will therefore be the first to examine the isolated effect of RT on ‘the self’. Hence, the purpose of this review was to systematically examine the effect of RT interventions on ‘the self’ in youth.

## Methods

The search strategy and inclusion criteria were specified and documented in advance on PROSPERO (number CRD42016038365). The conduct and reporting of this review adhered to the guidelines outlined in the PRISMA statement [23].

### Search Strategy

Electronic literature databases were searched from the year of their inception to October 2018. These were PubMed, MEDLINE, ERIC, PsycINFO, Embase, SPORTDiscus and Scopus. Relevant references from published literature were followed up and included where they met the inclusion criteria, and literature not identified in the electronic searches was sourced. This involved the use of ResearchGate to identify research papers written by key researchers in the field. Additionally, these researchers were contacted regarding any literature not yet published and the authors of the present review searched their personal libraries.

The search terms were related to ‘the self’, youths and RT (see Table 1). The Boolean operator ‘AND’ was used between categories, and the operator ‘OR’ was used within categories. The search strategy was adapted for each database, and searches were logged.

**Table 1:** Systematic review categories and search terms

Target population	Resistance training	‘Self’
youth*, young, child*, teen*, adol*, pube*, boys, girls	resistance training, resistance program*, resistance intervention, resistance exercise, weight training, strength and conditioning	self*, competenc*

*Search term truncated

Titles of potentially relevant articles were retrieved using the search strategy, duplicates then removed, and then the titles and abstracts were screened by one reviewer (HC). Ten percent (*n* = 71) of the titles and abstracts were screened by a second reviewer (JB). The inter-rater reliability for the two reviewers was found to be Kappa = 1.0 for both the screening and the quality assessment, suggesting a strong level of agreement [24]. Full-text copies were obtained for potentially eligible articles and assessed by two reviewers (HC and JB). During the review of full-text articles, a majority decision was taken in consultation with the other reviewers when disagreements regarding inclusion/exclusion occurred.

### Inclusion/Exclusion Criteria

Studies with participants of school age, between 5 and 18 years, were searched for. No studies were included where the subject group was identified as having a pathological condition or disability which affects movement, such as cerebral palsy or dyspraxia, and no studies were included where the subject group was identified as having a behavioural or neuropsychological condition such as autism or attention deficit hyperactivity disorder (ADHD). There may be differential adherence, impact and need for different programmes for groups of children with these identified conditions, so they were excluded from the searches. However, an avenue for future work could be to examine these groups but it was out with the scope of this review.

To allow an isolated review of RT, all included studies employed RT methods but were excluded if they contained plyometric, vibration or neuromuscular training, training specifically for rehabilitation purposes or additional activity (such as an aerobic component or games). Although some of these modes of training may also be viewed as forms of RT, this review aimed to investigate if isolated strength exercises alone had an effect on ‘the self’, as in particular plyometric training (power-based exercises) and rehabilitation exercises may be more relevant for a sporting population who may be aiming to improve performance. There was no restriction on location (e.g. school based or sports centre) or timing (e.g. during or after school).

Studies were included that used a control group and also those that did not, but all included a pre- and post-intervention measure.

### Data Extraction

Data were extracted using an electronic form by one reviewer (HC) and included study characteristics (e.g. country, year), participant characteristics (e.g. sample size, age, sex), intervention components (e.g. setting, duration, content) and changes in the outcomes (e.g. change in questionnaire score). For the participant characteristics, there is a difference between the sex of the training group (i.e. male only, female only or mixed-sex training group) and sex of the participant (male or female). This allows consideration of the impact of (for example) males training in a male-only versus a mixed training group environment. The outcome data were extracted in the form of mean, standard deviation and sample size. To check reliability, a second reviewer (JB) carried out data extraction on the first 10% of the included studies, which were in alphabetical order of the first author. Following this, any disagreements were resolved through discussion with all authors.

### Methodological Quality and Risk of Bias Assessment

The ‘Quality Assessment Tool for Quantitative Studies’ developed by the Effective Public Health Practice Project in 1998 was used to assess the quality and risk of bias of the included studies. The results of the assessment lead to an overall methodological rating of strong (identified as 1), moderate (identified as 2) or weak (identified as 3) in eight sections: selection bias, study design, confounders, blinding, data, collection methods, withdrawals and dropouts, intervention integrity and analysis. The assessment tool has been found to be valid and reliable [25].

To check reliability, JB carried out this assessment on all of the included studies. Following this, any disagreements were resolved through discussion between HC and JB.

### Meta-analysis

Random effects meta-analyses were conducted with the Comprehensive Meta-analysis software (version 2.2.064). Hedges’ *g* with randomised effects and 95% CIs were calculated for trials with sufficient data. The magnitude of Hedges’ *g* was interpreted using Cohen’s [26] convention as small (0.2), medium (0.5) and large (0.8). A significance level of *P* ≤ 0.05 was applied. Heterogeneity was assessed using the *I*^2^ statistic. For interpretation, *I*^2^ values of 25%, 50% and 75% were considered to indicate low, moderate and high heterogeneity, respectively [27]. Publication bias was assessed by calculating Egger bias statistics [28] and Rosenthal’s Fail-safe N [29]. Corresponding funnel plots were created.

### Moderator Analysis

A moderator analysis was conducted to determine whether the intervention effects on the outcomes differed by sex of participants (males or females), sex of training group (i.e. the training group was designed for either males, females or mixed sex), weight status (healthy weight, overweight/obese, obese or mixed weight status), age (< 12 or > 12 years, based on primary and secondary school age split), pubertal stage (< Tanner stage 2 or > Tanner stage 2, based on pre-pubertal and post-pubertal stages), location (school during physical education (PE), school during free time or community), type of control (no resistance training, nutrition input only, normal activity, PE) and quality of study (weak, moderate or strong). Although data were also extracted for frequency and duration of interventions, a moderator analysis was not conducted on these data due to the inappropriateness of separating their independent and combined impact on training outcomes (e.g. sessions being once a week for 12 weeks versus sessions being twice a week for 6 weeks).

## Results

**Fig. 2:**
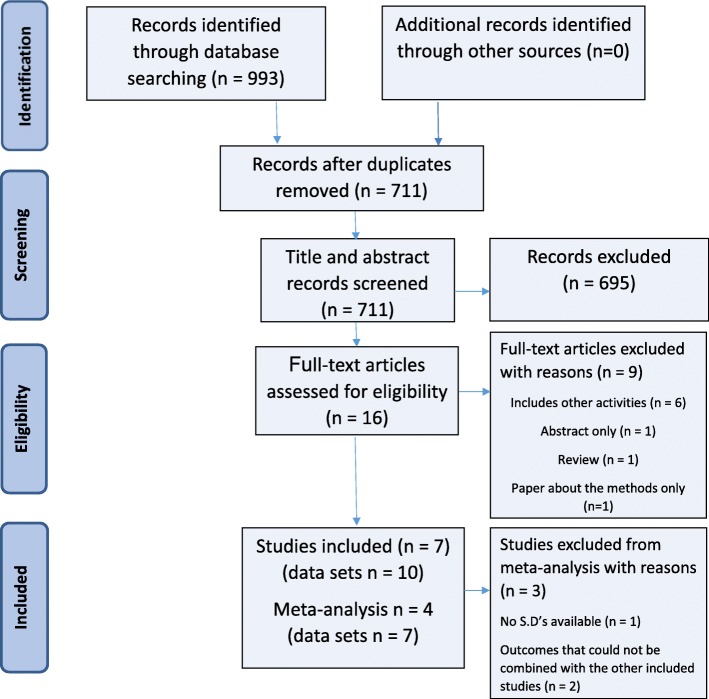
PRISMA flow diagram of systematic search and included studies

Out of an initial 993 studies identified through database searches after screening titles and abstracts, 16 studies were included (Fig. 2). Out of these 16 studies, nine were excluded from the meta-analysis and review as they did not meet the inclusion criteria. All of the included studies were peer-reviewed journal articles. Three studies met the inclusion criteria but were not included in the overall effect size estimates. This was because one did not provide standard deviations [30] and a further two did not assess outcomes that could be combined with the data from the four studies that were included (self-concept, upper body self-efficacy, lower body self-efficacy and exercise self-efficacy) [31, 32]. However, the findings from these three studies are included in Table 3 and the individual results are discussed. From the four studies that were included in the meta-analysis, seven sets of data were analysed (with some studies having more than one intervention group).

The study details included in the meta-analysis can be found in Table 2, and those not included in the cumulative effect size estimates are reported below and can be found in Table 3. Studies were conducted in three different countries (USA, Australia and Canada). In total, there were 256 participants in the experimental groups (sample sizes ranged from 13 to 78 participants) and 204 participants in the control groups (sample sizes ranged from 9 to 76 participants). The age of all participants ranged from 10 to 16 years, and only four studies reported Tanner stages (biological age) which ranged from 1 to 5.

**Table 2:** Description of included studies/data sets in the meta-analysis

Study	Country	Participant details						Intervention details						
		*N*	Age (years ± sd)	Pubertal stage (Tanner)	Weight status	% males	Sex of training group	Location	Weeks × per week (min per session)	Sets/reps/intensity	Exercises	Assessment tool	Outcomes included	Quality score
Goldfield et al. [33]	Canada	EG 78, CG 76	EG 15.9 ± 1.5, CG 15.6 ± 1.3	4–5	Obese	29%	Mixed	Community	22 weeks × 4 (45 min)	2–3 sets, 6–15 reps, moderate to max	Session 1: bench press, chest fly, lateral raise, shoulder shrugs, bicep curl, triceps press, abdominal crunches. Session 2: incline bench press, incline chest fly, shoulder press, front raise, preacher curl, assisted triceps dips, sit-ups. Session 3: squat, seated leg curl, front lateral pull down, seated row, lunge, straight leg raise, abdominal crunches. Session 4: leg press, leg extension, dumbbell pullover, seated row, lying leg curl	Physical Self-Perception Profile for Children [34] The Harter Global Self-Esteem sub-scale [35]	Body attractiveness, global self-esteem, physical condition, physical self-worth, physical strength, sport competence	1
Lubans et al. (a) [20]	Australia	EG 22, CG 14	EG 15.3 ± 0.8, CG 14.8 ± 0.4	/	Mixed	100%	Mixed	School—free time	8 weeks × 2 (50 min)	2 sets, 8–12 reps, max for reps	Free weights—squat, lunge, calf raise, bent over row, chest press, front raise, biceps curl, triceps extension, crunch and Russian twist	Children’s Physical Self-perception Profile [34], an adapted resistance training efficacy scale	Physical self-worth, sport competence, physical condition, body attractiveness, physical strength, RT self-efficacy.	2
Lubans et al. (b) [20]	Australia	EG 15, CG 16	EG 14.9 ± 0.6, CG 14.6 ± 0.6	/	Mixed	0%	Mixed	School—free time	8 weeks × 2 (50 min)	2 sets, 8–12 reps, max for reps	Free-weights—squat, lunge, calf raise, bent over row, chest press, front raise, biceps curl, triceps extension, crunch and Russian twist	Children’s Physical Self-perception Profile [34], an adapted resistance training efficacy scale	Physical self-worth, sport competence, physical condition, body attractiveness, physical strength, RT self-efficacy	2
Lubans et al. (c) [20]	Australia	EG 20, CG 14	EG 15 ± 0.6, CG 14.8 ± 0.4	/	Mixed	100%	Mixed	School—free time	8 weeks × 2 (50 min)	2 sets, 8–12 reps, max for reps	Elastic tubing—squat, lunge, calf raise, bent over row, chest press, front raise, biceps curl, triceps extension, crunch and Russian twist	Children’s Physical Self-perception Profile [34], an adapted resistance training efficacy scale	Physical self-worth, sport competence, physical condition, body attractiveness, physical strength, RT efficacy	2
Lubans et al. (d) [20]	Australia	EG 21, CG 16	EG 15 ± 0.7, CG 14.6 ± 0.6	/	Mixed	0%	Mixed	School—free time	8 weeks × 2 (50 min)	2 sets, 8–12 reps, max for reps	Elastic tubing—squat, lunge, calf raise, bent over row, chest press, front raise, biceps curl, triceps extension, crunch and Russian twist	Children’s Physical Self-perception Profile [34], an adapted resistance training efficacy scale	Physical self-worth, sport competence, physical condition, body attractiveness, physical strength, RT self-efficacy	2
Schranz et al. [36]	Australia	EG 30, CG 26	EG 14.9 ± 1.4, CG 15.1 ± 1.6	≥ 2	Overweight/obese	100%	Males	Community	24 weeks × 3 (75 min)	1–3sets, 8–12 reps, to concentric failure	Bench press, leg press, lat pulldown, leg curl (lying or seated), shoulder press (seated), seated row, biceps curl, triceps press-down, calf raise (seated) and abdominal crunch	Exercise Self-efficacy [37] Modified Resistance Training Self-efficacy and Outcome Expectancy Questionnaire [20], Physical Self Worth Scale [38], Self-Perception Profile for Adolescents [39]	Physical self-worth, global self-esteem, exercise self-efficacy, resistance training beliefs, resistance training confidence	1
Velez et al. [40]	USA	EG 13, CG 15	EG 16 ± 0.2, CG /	/	Mixed	53%	Mixed	School—free time	12 weeks × 3(40 min)	2–3 sets, 10–15 reps, 80% of 10 RM, RPE 10	Bench press, seated row, shoulder press, lat pulldowns, flies, bicep curls, and triceps pushdowns or lower body exercises including squats, Romanian dead lift, leg extensions, leg curls, lunges and calf raises	The CY-PSPP [34]	Body attractiveness, global self-esteem, physical condition, physical self-worth, physical strength, sport competence	2

Lubans et al. ‘a’ indicates male free-weights group, ‘b’ indicates female free-weights group, ‘c’ indicates male elastic tubing group, and ‘d’ indicates female elastic tubing group. “/” indicates no data available

*EG* experimental group, *CG* control group, *RT* resistance training, *sd* standard deviation, *rep* repetition, *max* maximum, *RM* repetition maximum, *RPE* rate of perceived exertion, *lat* latissimus dorsi, *CY-PSPP* Children and Youth - Physical Self-Perception Profile

**Table 3:** Studies not included in the cumulative effect size estimates

Study	Country	Participant details						Intervention details						
		*N*	Age (years ± sd)	Pubertal stage (Tanner)	Weight status	% males	Sex of training group	Location	Weeks × per week (min per session)	Sets/reps/intensity	Exercises	Assessment tool	Outcomes	Quality score
Faigenbaum et al. [31]	USA	EG 24, CG 9	EG 10.8 ± 0.4, CG 10 ± 0.4	1–2	Healthy	46	Mixed	Community	8 weeks × 2 (60 min)	2–3 sets, 6–8 reps, 6 RM	Leg extension, chest press, leg curl, overhead press, bicep curl	The Martinek Zaichkowsky Self-concept Scale for Children [41] Adapted Self-efficacy Scale [42]	Self-concept, self-efficacy upper body, self-efficacy lower body	3
Holloway et al. [30]	USA	EG 13, CG 18	EG 16, CG 16	/	Healthy	0	Females	School—free time	12 weeks × 3 (60 min)	3 sets, 3–10 reps, 65–80% 1 RM	Free weight Olympic-style parallel back squat, bench press, behind-the-neck standing shoulder press, bent-over rows, abdominal crunches	The Physical Self-efficacy Scale [43]	Total self-efficacy, weight training efficacy, physical task efficacy, perceived physical ability, physical self-presentation, physical self-efficacy	2
Mullane et al. [32]	USA	EG 20	EG 10.5 ± 0.5	1	Mixed	90	Mixed	Community	8 weeks × 2 (45 min)	Circuit 60–90s, 6 exercises	Leg press, assisted pull ups, shoulder press, bicep curls, pushups, sit ups	Adapted Children’s Self-efficacy Scale [44]	Self-efficacy of exercise	2

“/” indicates no data available

*EG* experimental group, *CG* control group, *sd* standard deviation, *rep* repetition, *max* maximum, *RM* repetition maximum

The training programmes varied in duration from 8 to 24 weeks (mean 13.4 ± 6.8 weeks) with a mean training frequency of 2.7 ± 0.76 sessions per week (2–4 times a week) and session duration of 53.6 ± 12.1 min (ranging from 45 to 75 min). The average attendance reported by the studies that reported it was 78%. All but one study (which was a circuit-based intervention) included 1–3 sets, with reps ranging from 3 to 15 of a moderate to maximum intensity.

Through the quality assessment process for all of the studies included, two of the studies were classified as ‘strong’, four of the studies were classified as ‘moderate’ and one study was classified as ‘weak’. See Tables 2 and 3 for details.

### Meta-analysis

**Fig. 3:**
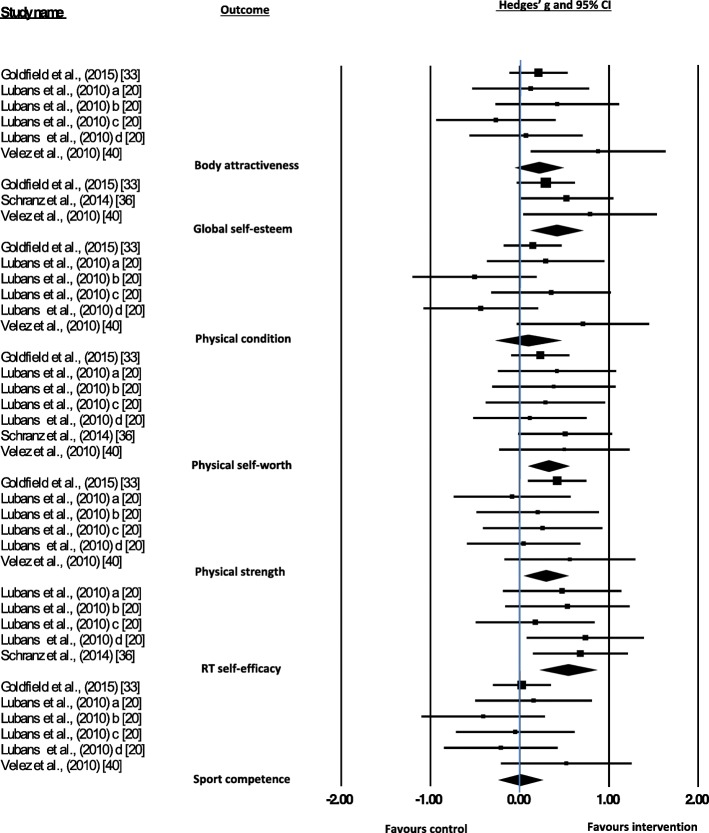
Forest plot of ‘the self’ meta-analyses. Lubans et al. ‘a’ indicates male free-weights group, ‘b’ indicates female free weights group, ‘c’ indicates male elastic tubing group, and ‘d’ indicates female elastic tubing group

Four studies (including seven data sets) were evaluated in a meta-analysis. Seven outcome variables related to ‘the self’ were included in the meta-analysis: resistance training self-efficacy, perceived physical strength, physical self-worth, global self-worth, perceived body attractiveness, perceived physical condition and sport competence. Figure 3 illustrates the effect sizes for all of the data sets and the overall effect size for each outcome: resistance training self-efficacy (Hedges’ *g* = 0.538, 95% CI 0.254 to 0.822, *P* < 0.001), physical strength (Hedges’ *g* = 0.289, 95% CI 0.067 to 0.511, *P* = 0.011), physical self-worth (Hedges’ *g* = 0.319, 95% CI 0.114 to 0.523, *P* = 0.002), global self-worth (Hedges’ *g* = 0.409, 95% CI 0.149 to 0.669, *P* = 0.002), body attractiveness (Hedges’ *g* = 0.211, 95% CI − 0.031 to 0.454, *P* = 0.087), physical condition (Hedges’ *g* = 0.089, 95% CI − 0.238 to 0.417, *P* = 0.593) and sport competence (Hedges’ *g* = 0.004, 95% CI − 0.218 to 0.225, *P* = 0.974). Significant effect sizes were observed for resistance training efficacy, physical strength, physical self-worth and global self-worth, and although not significant, there was a small effect size for body attractiveness. There were inconclusive findings for physical condition and sport competence. Based on the thresholds categorised by Higgins and colleagues [27], low to medium heterogeneity was identified for all outcomes (i.e. *I*^2^ = 0–44.9%) and so moderator analysis was undertaken.

### Moderator Analysis

Moderator analysis was conducted to determine whether the intervention effects on each outcome variable differed by sex of participants, sex of the training group, weight status, age, pubertal stage, location, type of control group and the quality score of the studies. There was a selected impact of sex on perceived physical condition (*Q*(df) = 6.994(2), *P* = 0.030) with two studies including females (Hedges’ *g* = − 0.469, 95% CI − 0.942 to 0.005, *P* = 0.052), two studies including males (Hedges’ *g* = 0.0322, 95% CI − 0.148 to 0.792, *P* = 0.180) and two studies that were mixed sex (Hedges’ *g* = 0.323, 95% CI − 0.188 to 0.835, *P* = 0.216). There were no other significant moderation effects. However, there was a large significant effect size for the one study that included PE as a control group for body attractiveness, although the effect size did not differ statistically from other types of control groups.

### Individual Study Results

For the studies that were not included in the meta-analysis, Faigenbaum et al. [31] reported no statistically significant increases in comparison with the control group for the outcomes of self-concept (*P* = 0.09), lower body self-efficacy (*P* = 0.69) and upper body self-efficacy (*P* = 0.87). Holloway et al. [30] included total self-efficacy, weight training efficacy, physical task efficacy, perceived physical ability, physical self-presentation and physical self-efficacy and reported statistically significant increases in all of the outcomes compared to the control group (*P* < 0.05) apart from weight training efficacy (*P* = 0.22), although they did report a significant increase over time for the intervention group (*P* = 0.04). For pre- and post-intervention measures, Mullane et al. [32] reported a statistically significant increase in self-efficacy of exercise over time (*P* = 0.008).

### Publication Bias

**Fig. 4:**
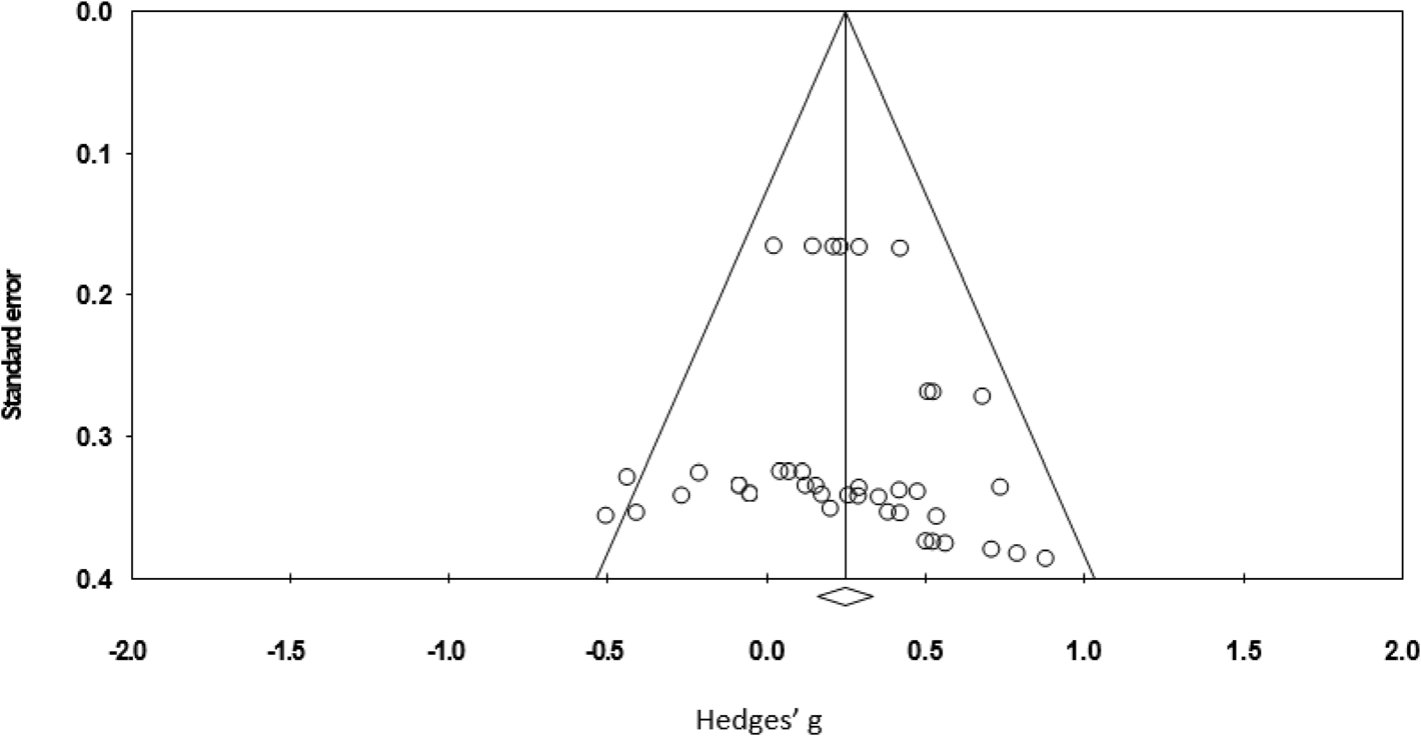
Funnel plot to show study publication bias

To identify possible publication bias, effect sizes were plotted against standard errors to yield the funnel plot shown in Fig. 4. This indicated that there was no presence of publication bias and this was confirmed by a non-significant result from Egger’s test [28]. Rosenthal’s fail-safe N [29] found that 258 additional studies would be needed for the cumulative effect to be non-significant.

## Discussion

The UKSCA’s and NSCA’s position statements on youth RT both suggest that RT may have a positive impact on ‘the self’ [17, 18]. This is the first meta-analysis to synthesise the existing literature and provide robust initial support for these statements, and the results of this meta-analysis support a positive impact of RT on ‘the self’ in youth.

In summary, there was a statistically significant medium effect size of RT interventions on resistance training self-efficacy and small significant effect sizes for physical strength, physical self-worth and global self-worth. Although not significant, there was a small effect size evident for body attractiveness. There were inconclusive findings for the effect on physical condition and sport competence.

### Outcomes

To investigate each of the outcomes, starting with resistance training self-efficacy, the meta-analysis suggests a positive intervention effect. This positive association between RT and resistance training self-efficacy was a logical finding based on the nature of the intervention and fits with suggestions based on the EXSEM model [11] that the most immediate and specific aspects of the self are likely to be influenced by targeted interventions. Comparably, for the outcome of perceived physical strength, the meta-analysis indicated a small but significant effect size, which again would be a logical finding given the nature of the intervention.

There were small significant effect sizes evident for physical self-worth and global self-worth. In exploring the possible mechanism behind all of the significant findings, the EXSEM model [11] may provide an explanation. The model (Fig. 1) proposes that perceived physical competencies developed through exercise, including RT, can enhance global self-esteem. This would support the current finding that RT had a positive effect on physical self-worth, physical strength and resistance training efficacy which subsequently may have also had a positive effect on global self-worth. Additionally, the findings of this review are also consistent with the EXSEM model [11], in that the outcomes that correspond most to the intervention (perceived strength, resistance training efficacy and physical self-worth) displayed significant effect sizes. In further support of this, there is previously published review-level evidence that provides support for a positive relationship between PA and constructs relating to ‘the self’ [7–10]; however, these authors did suggest that because findings varied by methodological design and quality, additional randomised controlled trials should be conducted to replicate and confirm the findings. Isolated RT was also not explored within these reviews.

Furthermore, the conceptual model proposed by Lubans et al. [14] suggests that physical activity has an impact on psychosocial factors (i.e. social connectedness, mood and emotions and physical self-perceptions) which ultimately has an effect on global self-esteem. The findings of the current review support this model with RT having a positive impact on physical self-perceptions which then further impact on the more global measure.

There were additional outcomes that did not display significant effect sizes. When investigating body attractiveness, the meta-analysis showed a small but not significant effect of RT, and the inconsistency in the results of the individual studies may explain this finding as may the inclusion of males and females. For example, Lubans et al. [20] found a significant increase in perceived body attractiveness in females, but not in males. For physical condition, the current meta-analysis indicated no conclusive effect of the interventions. This construct is defined as the perceived level of physical conditioning and exercise, and therefore, it may be argued that it reflects aerobic conditioning, and as such, RT might not be expected to exert any influence. Similarly, for sport competence, the meta-analysis does not support a positive effect of the interventions. As the RT content of the studies in this review is not sport specific, this is likely to explain the findings, particularly when the participants are not identified as being involved in organised sport.

From the additional studies that were not included in the meta-analysis, there were findings that support a positive effect of RT on other constructs of ‘the self’.

Mullane et al. [32] investigated the impact of RT on exercise self-efficacy and found that there was a significant increase over time based on pre-/post-intervention measures. Holloway et al. [30] explored the effect of resistance training on total self-efficacy, weight training efficacy, physical task efficacy, perceived physical ability, physical self-presentation and physical self-efficacy, and interestingly, the only outcome that did not significantly increase in comparison with the control group was weight training efficacy, which conflicts with findings of the current meta-analysis, but these investigators reported that the groups were not randomly assigned, which may have had an impact on the findings.

Similarly, Faigenbaum et al. [31] examined the effect of RT on self-concept, upper body self-efficacy and lower body self-efficacy and reported no substantial improvement. However, it was identified that this could be due to ceiling effects. Furthermore, these authors identified a limitation of their study being that the children may not have understood the questionnaire due to their young age (pre-adolescent) [31]. If this is correct, it may be a limitation that is applicable to other studies.

In summary, the evidence suggests that there is a positive effect of RT on specific measures of ‘self’, but not for the more general measure of self-concept, which in relation to the EXSEM model [11] is a logical finding given that self-concept is the apex of the hierarchy, therefore more stable and less likely to change.

### Moderator Analysis

To investigate the findings further, a moderator analysis was completed on all outcomes to identify if any effects could be explained by specific moderator variables. Sex was the only significant moderator intervention effect. Of note, resistance training intervention effects on perceived physical condition were significantly larger in boys compared to girls and mixed-sex groups. There is no explanation offered in the individual studies included in this analysis regarding male and female differences in this context. However, with perceived physical condition relating to aerobic conditioning, it might not be expected to improve due to the nature of the intervention. Thus, the finding for males is surprising, although Hayes et al. [45] reported that males had significantly higher levels of physical self-perceptions than females. It should be noted that there was also a large significant effect size for the one study that included PE as a control group for body attractiveness, but as this was just involving one study, it is difficult to make conclusions based on these data.

It is important to note that there was considerable variability within studies and if there had been a greater number of studies/more equal in comparison that a robust moderator analysis may have been possible. Additionally, although we did not include duration or frequency in the moderator analysis, it should be noted that there was a wide range of duration, with two studies including an intervention of over 20 weeks [33, 36]. These studies did report significant effects, but also were the only studies to include only overweight/obese participants, which may also have had an impact on the findings.

Although the intervention content described by the included studies is in line with the recommendations from the NSCA and the UKSCA [17, 18], there was still considerable disparity between participant numbers and the intervention content of the studies. It is not possible to indicate whether effects were influenced by training frequency, volume or intensity or study duration, but it is likely that these would be influential and this should be considered in future research.

Although the moderator analysis did not suggest this, the variability between studies could also be accounted for by the mixed weight status of participants since the current meta-analysis included overweight and obese participants. There have been findings to suggest that those who are overweight or obese may have lower baseline ‘self’ values than their healthy weight counterparts [46] and therefore less chance of ceiling effects. Contrary to this, overweight pre-adolescents may also have higher perceived strength than their healthy weight peers [47]. Therefore, there are implications of the possible impact RT could have on ‘the self’ specifically in an overweight/obese population. As the current meta-analysis included both healthy weight and overweight/obese participants, this variation based on weight status could have had an effect on the reported outcomes and should be considered when interpreting these results and when designing further studies.

### Strengths and Limitations

There were a number of strengths to this review. There should be strong confidence in the main findings given the rigorous review process, including a pre-published protocol. There were a strict inclusion/exclusion criteria which resulted in a review of 7 studies that examined the effects of RT on ‘the self’ in 460 youths from 3 countries. Despite the small number of studies, this is the first review to investigate the impact of RT on ‘the self’. Furthermore, there was low heterogeneity showing that few differences were identified across the studies allowing for appropriate pooling of the data, and additionally, there was no evidence of publication bias so there is less chance of an overoptimistic conclusion.

There was high compliance reported in the included studies (an average of 78% for the four studies that reported it), which is important because it increases confidence that the effects were due to the intervention. Additionally, good compliance is important given that exercise adherence is critical to positive outcomes [48], adding substance to the potential for RT as a viable mode of intervention to improve outcomes related to ‘the self’.

Despite the strengths of the review, there are limitations that need to be considered when interpreting the results. Although there were positive effects reported in this review, there were only a small number of studies included and there were variable findings between the studies making the generalisability of the results difficult. Four of the seven studies included were from the USA, and the seven studies examined varied significantly, including gender, weight status and study design. Additionally, although some of the included studies analysed male and female data separately, some have combined the data. This introduces limitations due to variability in adaptations to RT interventions according to sex [49]. Furthermore, the moderator analysis did not include duration or frequency due to the inappropriateness of separating their independent and combined impacts on training outcomes.

A range of assessment tools was used to measure different parameters of ‘the self’ which has implications for the validity of the meta-analysis. However, the majority of the studies included used the Children and Youth Physical Self-perception Profile (CY-PSPP) which has been validated as a method of assessing physical self-perceptions in children and youth [50]. Finally, there was a mixture of quality of the studies included, with two of the studies classified as ‘strong’, four classified as ‘moderate’ and one classified as ‘weak’. More high-quality studies are therefore required to investigate this topic in more depth.

Given the small number of studies analysed, it is difficult to provide practical implications for developing an effective intervention. However, as all of the interventions included in this review were in line with the UKSCA and NSCA recommendations, with the content favouring whole body RT (including elastic bands, free weights, machine weights or body weight) of 1–3 sets and 3–15 reps of moderate to maximum intensity, reference to these position statements for guidance is recommended. Additionally, as there were limited significant findings from the moderator analysis, caution should be exercised over using these results to inform study design.

## Conclusions

This review of the literature suggests that RT may have a positive effect on ‘the self’ in youth, although more high-quality research is required to substantiate this. This meta-analysis provides an overview of the current research evidence and an insight into the potential benefits of such interventions.

The meta-analysis found a statistically significant, medium effect of RT interventions on resistance training efficacy and small effects on global self-worth, physical self-worth and perceived physical strength. There was a non-significant small effect size for body attractiveness and inconclusive effects on physical condition and sport competence. While we can conclude that RT interventions have a positive impact on ‘the self’, it is noted that this reflects only a small body of published work.

With RT interventions offering potential benefits for youth with regard to ‘the self’, it is imperative that more robust and high-quality studies should be conducted to further investigate the role RT may play in the enhancement of ‘the self’. The findings of this review support the inclusion of RT when developing PA intervention strategies due to the positive association between ‘the self’ and PA levels. Based on the findings of this meta-analysis and review, future studies should be designed as randomised controlled trials with large samples and include a treatment group with an isolated RT intervention. There should be careful consideration given for appropriate intervention content and assessment methods. If validated, this type of intervention, as recommended by the UK and WHO PA guidelines, could ultimately have a positive impact on ‘the self’ and improve the health of individuals not only during childhood but as they progress through life.

## Data Availability

After publication, all data necessary to understand and assess the conclusions of the manuscript are available to any reader of Sports Medicine-Open.

## Abbreviations

BASES: British Association of Sport and Exercise Sciences
EXSEM: Exercise and Self-esteem Model
NSCA: National Strength and Conditioning Association
PA: Physical activity
PSPP: Physical Self-perceptions Profile
RT: Resistance training
UKSCA: United Kingdom Strength and Conditioning Association
